# Grandparenting, intergenerational coparenting, and mental health of vulnerable children in Chinese families: risks, resilience, and clinical implications

**DOI:** 10.3389/fpsyg.2026.1934788

**Published:** 2026-09-16

**Authors:** Guangzhe Frank Yuan, Aihua Su

**Affiliations:** 1 School of Education Science, Leshan Normal University, Leshan, China; 2 School of Nursing, Shandong First Medical University (Shandong Academy of Medical Sciences), Tai’an, Shandong Province, China

**Keywords:** child mental health, family systems, grandparenting, intergenerational coparenting, resilience

## Abstract

In China, grandparents serve as primary or co-caregivers for millions of children, particularly those separated from rural-to-urban migrant parents. This short communication synthesizes evidence on how grandparenting quality and intergenerational coparenting dynamics shape the mental health outcomes of vulnerable Chinese children, including those in left-behind, three-generation urban, and dual-income family configurations. We distinguish throughout between children whose parents have both migrated (leaving grandparents as sole custodial caregivers) and children who remain in triadic parent-grandparent–child systems, whether through partial parental migration or co-resident urban caregiving arrangements, as these configurations carry differing implications for grandparenting quality and coparenting dynamics. Evidence from cross-sectional surveys, meta-analytic studies, and dyadic interdependence models indicates that positive grandparenting predicts fewer child behavioral difficulties and greater prosocial competence, whereas coparenting conflict, low coparenting relationship quality, and caregiver depressive symptoms constitute compounding risk pathways. Grandparental warmth, harmonious coparenting, and family resilience are identified as protective mechanisms within a family systems framework. Clinical and nursing implications are discussed, with focus on intergenerational interventions that simultaneously engage grandparents and parents. The present paper argues that mental health professionals and nursing practitioners working with Chinese children should adopt a multigenerational assessment lens, given that standard clinical protocols focused on the parent–child dyad may systematically underestimate intergenerational risk and resilience processes. Culturally adapted, evidence-based coparenting programs represent a priority area for intervention development in Chinese psychiatric and nursing contexts.

## 1 Introduction

China’s rapid urbanization and large-scale rural-to-urban labor migration have profoundly restructured family caregiving arrangements. Both trends converge on a single underlying phenomenon: the substantially increased centrality of grandparents in Chinese children’s daily caregiving, whether as the primary or sole custodial caregiver for children of migrant parents, or as a co-resident, shared caregiver alongside present parents. Nationally representative monitoring data indicate that approximately 69.7 million rural left-behind children (LBC) reside in villages while one or both parents have migrated for urban employment (Tong et al., 2019). These LBC may have access to fewer resources and are ten times as likely to fall into poverty as their non-LBC counterparts (Yu et al., 2025). Importantly, LBC are not a homogeneous group with respect to caregiving structure: following the classification used by Akezhuoli et al. (2022), children whose both parents have migrated (BLBC) are typically raised in a dyadic grandparent–child unit in which the grandparent functions as sole custodial caregiver, whereas children with only one migrated parent (MLBC or FLBC) remain embedded in a triadic parent-grandparent-child system in which coparenting dynamics between the resident parent and grandparent continue to operate. Urban three-generation households, in which grandparents share childcare with resident parents, are equally prevalent; approximately 66.5% of Chinese grandparents are actively involved in raising grandchildren [Li et al., 2016, as cited in Chen J. et al. (2024) and Chen S.-M. et al. (2024)]. This arrangement, termed intergenerational coparenting, refers to the joint exercise of caregiving responsibilities between parents and grandparents within the family system (Bai et al., 2023). Taken together, LBC (particularly MLBC/FLBC) and urban three-generation households represent two structurally distinct but conceptually related pathways through which grandparental involvement has intensified in contemporary Chinese families, and it is this shared theme of intensified grandparental involvement—operating either as sole custodial care or as shared coparenting—that motivates the present synthesis.

Both grandparenting quality and the nature of the parent-grandparent coparenting relationship carry substantial implications for child mental health and development. In clinical and nursing practice, children presenting with internalizing problems (e.g., anxiety, depression, withdrawal) or externalizing problems (e.g., conduct difficulties, aggression) are frequently embedded in complex multigenerational caregiving contexts that remain inadequately assessed. The mental health risks and resilience resources operating within these contexts are insufficiently integrated into current clinical frameworks, particularly in psychiatry and nursing settings serving Chinese populations. Because the applicability of grandparenting and coparenting mechanisms differs across custodial (grandparent-only) and triadic (parent-present) configurations, we consider these two configurations separately where the evidence permits. The present article therefore aims to (1) synthesize the principal risk and resilience pathways linking grandparenting and intergenerational coparenting to child mental health outcomes, and (2) derive evidence-informed clinical and nursing practice implications.

## 2 Custodial grandparenting and child mental health: evidence from left-behind children

The relationship between grandparenting and child mental health is neither uniform nor straightforwardly protective. Research on LBC has consistently documented elevated rates of internalizing difficulties relative to children who reside with both parents. Parental migration has been associated with significantly adverse health-related outcomes in LBC adolescents across multiple domains, including mental health, health behaviors, and quality of life (e.g., Akezhuoli et al., 2022; Wang et al., 2023). A recent systematic review of 35 studies found that Chinese rural LBC are disproportionately affected by depression, social anxiety, internet addiction, and suicidal ideation relative to non-left-behind children, with these conditions sharing overlapping risk factors and demonstrating significant co-occurrence (Li J. et al., 2026). A large-scale study in southwest China found a 4.22% lifetime and 3.84% current prevalence of clinically diagnosed depressive disorders in LBC, with depressive disorders strongly associated with self-harm behaviors, particularly among girls and younger children (Ran et al., 2024). Wang et al. (2019) demonstrated that both current and prior parental migration were associated with significantly elevated mental health difficulties, including emotional symptoms, conduct problems, and hyperactivity, even after adjustment for confounders. It is important to note that none of the studies cited in this paragraph directly measured grandparental involvement, grandparenting quality, or grandparent caregiving behavior as a predictor variable; grandparents are implicated only indirectly, as the default custodial caregivers for children whose parents have migrated. These studies therefore establish that altered family caregiving structure (indexed by LBC status) is associated with poorer child mental health, but they do not test whether grandparenting quality itself accounts for this association—a gap addressed by the studies discussed below.

Directly relevant to this gap, a systematic review and meta-analysis of 38 studies encompassing 344,860 children found that children cared for by grandparents exhibited worse mental health status overall, including more internalizing problems, externalizing problems, and poorer socioemotional wellbeing, relative to parent-cared children, although effect sizes were generally small (Wang et al., 2024a,b). This meta-analytic evidence indicates that grandparental care as a structural arrangement carries measurable, if modest, mental health costs on average—underscoring the clinical importance of moving beyond structural caregiving categories to assess the quality of grandparenting directly, as detailed below.

Grandparenting practice does not merely substitute for parental care in a neutral fashion. Positive grandparenting, characterized by warmth, responsiveness, and behavioral oversight, has been associated with more prosocial behavior and fewer difficulties among young LBC (Liang and Van Leeuwen, 2025). This finding derives from a sample of young rural LBC and specifically measures grandparenting quality (rather than mere custodial status) as a predictor, in contrast to the studies cited in the preceding paragraphs. Conversely, overindulgent or inconsistent grandparenting, a pattern documented in Chinese custodial arrangements—referring specifically to skip-generation households in which grandparents serve as full-time, day-to-day custodial caregivers, most commonly in the context of BLBC whose both parents have migrated—has been linked to elevated emotional and behavioral problems (Li et al., 2019), whose study of grandparental parenting styles found that grandparental “Care” and “Overprotection” dimensions were positively associated with children’s emotional and behavioral problems. The clinical distinction is important: the mere presence of a grandparent as caregiver does not confer protection; it is the quality and consistency of grandparental practices that determine developmental outcomes. Grandparents who maintain warmth and appropriate behavioral monitoring function as a significant resilience resource, buffering children against the psychological costs of parental absence (Liang and Van Leeuwen, 2025).

Perceived parental unavailability, rather than physical absence alone, functions as a key mediating factor linking parental migration to child mental health, suggesting that children’s subjective experience of emotional abandonment carries greater psychological weight than objective caregiving arrangements (Wang et al., 2019). This construct should be distinguished from grandparental availability: perceived parental unavailability reflects the child’s subjective appraisal of the migrated parent’s emotional accessibility, whereas grandparental availability reflects the physical and emotional presence of the custodial or co-resident grandparent. These two constructs are not interchangeable and may in fact interact—a child may perceive low parental availability while simultaneously experiencing high grandparental availability, or vice versa. Evidence from Liang and Van Leeuwen (2025) suggests that grandparental warmth can partially offset the psychological impact of perceived parental unavailability, but does not fully substitute for it, implying that clinical assessment should capture both constructs independently rather than treating grandparental presence as a proxy for adequate emotional caregiving. This distinction has practical significance: structural indicators of caregiving configuration are insufficient for clinical assessment; practitioners must also explore children’s and caregivers’ appraisals of relational availability and psychological closeness.

In summary, the evidence reviewed in this section derives predominantly from rural LBC samples (Akezhuoli et al., 2022; Li J. et al., 2026; Ran et al., 2024; Wang et al., 2019; Liang and Van Leeuwen, 2025; Li et al., 2019), with the meta-analytic evidence of Wang et al. (2024a,b) drawing on a broader international pool of studies that includes but is not limited to Chinese samples. Direct evidence on grandparenting quality in urban three-generation households, where parents remain present, is comparatively limited and is addressed further in Section 3.

Theoretically, the studies reviewed in this section draw on somewhat different frameworks than those discussed in Section 3. Rather than a coparenting-systems lens, LBC-focused research has more often been grounded in the Intergenerational Solidarity Framework, which conceptualizes the grandparent-grandchild bond along affectual, associational, consensual, functional, normative, and structural dimensions of solidarity, and has been used to explain the protective and companionship functions grandparents serve for children of migrant parents (Wang et al., 2024a,b). A second theoretical driver in this literature is attachment theory, which frames perceived parental and grandparental availability as regulatory anchors for children’s emotional security, even across physical distance (Brumariu, 2015; Liang and Van Leeuwen, 2025). This contrasts with the family systems and coparenting-process framework (Feinberg, 2003; Minuchin, 1988) that predominates in the three-generation household literature reviewed in Section 3, which conceptualizes grandparents primarily as coparenting-system participants rather than as solidarity or attachment figures per se. We note this theoretical divergence explicitly because it may itself explain why the two literatures have developed largely in parallel, with limited cross-fertilization of measures or models—a gap we return to in Section 6.

## 3 Intergenerational coparenting: mechanisms of risk and protection

The coparenting framework introduced in this section applies most directly to family configurations in which a parent remains an active, present coparenting partner alongside the grandparent—that is, urban three-generation households and MLBC/FLBC families in which one parent has migrated but the other parent remains resident, actively engaged in decision-making with the grandparent. This distinction is important because the coparenting constructs discussed below (division of labor, mutual support/undermining, joint management) presuppose an ongoing, present coparenting partner. For BLBC in which both parents have migrated, “coparenting” may instead operate remotely—through periodic phone or video-mediated involvement in childrearing decisions—or may be functionally absent, with the grandparent serving as sole decision-maker. It is important to clarify, however, that even in BLBC families, parental involvement is rarely reduced to zero: migrant parents commonly remain involved through remittances, periodic communication, and participation in childrearing decisions from a distance, and this remote involvement has been shown to interact meaningfully with grandparental caregiving. Using a sample of 309 LBC cared for by grandparents, Chen J. et al. (2024) and Chen S.-M. et al. (2024) found that both father involvement and mother involvement—each measured directly despite the parents’ physical absence—predicted children’s emotional well-being through the mediating pathway of needs satisfaction, and that grandparent involvement moderated the effect of mother involvement specifically, functioning as a complementary rather than substitutive caregiving resource. This finding indicates that “coparenting from a distance” between a migrated parent and a custodial grandparent is empirically observable and consequential for child outcomes, even in the absence of a co-resident parent, and should not be conflated with a total absence of parental coparenting input. We flag this as an important boundary condition on the applicability of Feinberg’s (2003) framework to the full spectrum of vulnerable Chinese children considered in this paper, and note that theoretical extensions of coparenting constructs to distance-spanning caregiving arrangements remain underdeveloped (Bai et al., 2023).

Drawing on Feinberg’s (2003) foundational framework, intergenerational coparenting encompasses four interdependent components: agreement on child-rearing principles, division of child-related labor, mutual support or undermining of the coparenting partner’s role, and joint management of family interactions. When extended to three-generation families, this framework highlights additional complexity arising from generational differences in values, educational philosophies, and role expectations (Bai et al., 2023).

The scoping review by Bai et al. (2023), guided by family systems theory and an intersectionality perspective, identified five key dimensions of intergenerational coparenting: power and authority, division of labor, conflict, coping and adaptation, and reciprocity. Coparenting conflict emerged as a principal risk factor across studies. A meta-analysis of 93 studies involving 41,207 participants confirmed that coparenting conflict, competitiveness, and triangulation were each significantly associated with children’s internalizing and externalizing problems, with effect sizes ranging from *r* = −0.16 to −0.17, where negative values indicate that greater coparenting conflict predicts greater child difficulties, and these associations remained robust across diverse samples (Zhao et al., 2022). This meta-analysis, like most of the coparenting literature reviewed in this section, draws predominantly on urban, parent-present, three-generation family samples rather than LBC samples. In the Chinese context specifically, a recent mediation analysis with 426 participants demonstrated that intergenerational coparenting conflict was associated with increased child behavioral difficulties through the mediating role of family environment quality (Li Z. et al., 2026).

A particularly important mechanism concerns the transmission of caregiver mental health through the coparenting relationship to parenting behaviors and child outcomes. Using an actor-partner interdependence mediation model with 464 urban Chinese coparenting families, Chen J. et al. (2024) and Chen S.-M. et al. (2024) demonstrated that parents’ and grandparents’ depressive symptoms were each indirectly associated with harsher discipline and reduced supportiveness toward children, with associations fully or partially mediated by each party’s perceived coparenting relationship quality. Partner effects were also present: a parent’s depressive symptoms predicted grandparental harsh parenting through the grandparent’s own perception of the coparenting relationship, and vice versa. This sample consisted exclusively of urban three-generation families with resident parents; the applicability of these mediational pathways to MLBC/FLBC coparenting-at-a-distance arrangements has not, to our knowledge, been directly tested. These findings underscore the interdependence of caregivers in Chinese families and the potential for cascading harm when mental health difficulties emerge in any member of the intergenerational caregiving network.

A latent profile study by Xie et al. (2026) further clarified that mother-grandparent coparenting relationships cluster into distinct profiles, namely Discordant (29.2%), Balanced (46.7%), and Harmonious (24.0%)—these are the formal profile labels defined by the original authors and are capitalized here on first use to preserve fidelity to the source terminology; subsequent generic references use lowercase—with mothers in the Discordant group reporting the highest stress, most severe depressive symptoms, and poorest sleep quality. A study by He et al. (2024) found that the effects of coparenting relationship quality on preschoolers’ social competence differed by grandmother lineage, with maternal grandmothers’ coparenting perceptions serving as a stronger predictor than paternal grandmothers’, reflecting the deep imprint of Confucian filial piety norms on intergenerational family functioning in China (Yeh and Bedford, 2003).

In sum, the intergenerational coparenting evidence reviewed in this section is drawn almost entirely from urban, parent-present, three-generation family samples (Bai et al., 2023; Zhao et al., 2022; Li Z. et al., 2026; Chen J. et al., 2024; Chen S.-M. et al., 2024; Xie et al., 2026; He et al., 2024), in contrast to the predominantly LBC-derived evidence reviewed in Section 2. We are not aware of published studies that directly test whether these coparenting risk mechanisms operate similarly in MLBC/FLBC triadic families, where the parent’s participation in coparenting is constrained by physical distance; this represents an important gap for future comparative research, discussed further in Section 6.

## 4 Resilience pathways: toward a strength-based understanding

Despite well-documented risk mechanisms, the literature equally supports identification of protective pathways. Family systems theory posits that positive dynamics within one family subsystem can buffer adversity in another (Minuchin, 1988). In three-generation coparenting families, three resilience pathways merit particular clinical attention.

Grandparental involvement has been shown to directly and indirectly promote young children’s resilience. A structural equation modeling study found that grandparents’ involvement significantly predicted children’s resilience, with this association partly mediated by reduced maternal parenting stress and strengthened family cohesion (Wu et al., 2025). This study sampled urban, parent-present families; a related study of preschoolers in Chinese three-generation households similarly found that grandparent involvement was associated with reduced parenting stress and better child social adjustment, reinforcing the generalizability of this pathway within triadic caregiving configurations specifically (Luo et al., 2020). These findings indicate that engaged grandparenting reduces burden on mothers, creating a more emotionally stable caregiving environment that supports adaptive child functioning.

Coparenting harmony constitutes a second potent protective resource. In the latent profile study described above, mothers in Harmonious coparenting relationships reported the most favorable psychosocial outcomes across all measured domains, while stronger spousal coparenting and grandparental caregiving involvement were associated with membership in this class (Xie et al., 2026). A study of 1,279 Shanghai mothers found that family resilience moderated the relationship between unsupportive coparenting and children’s problem behaviors, such that the detrimental effect of coparenting conflict on child behavioral difficulties was significantly attenuated under conditions of high family resilience (Zhu et al., 2024).

Parent–child communication quality represents a third compensatory resilience mechanism in LBC families. Wang et al. (2019) reported that difficulties communicating with parents were strongly associated with total mental health difficulties among LBC, implying that maintained quality contact, even remotely via mobile technology, buffers the adverse psychological effects of parental absence. From an attachment perspective, perceived parental accessibility, even across physical distance, functions as a regulatory anchor sustaining children’s emotional security (Brumariu, 2015). This mechanism has been documented specifically in LBC samples; it remains an open empirical question whether an analogous communication-based resilience pathway operates in grandparent-custody, non-LBC configurations, or in three-generation households where parents are present but grandparents serve as the primary day-to-day caregiver. Indirect support comes from studies of parent-present, three-generation households, where grandparent–child and grandparent-parent communication quality (rather than distal parent–child communication specifically) has been linked to reduced parenting stress and improved child social adjustment (Wu et al., 2025; Luo et al., 2020), suggesting that communication-based resilience mechanisms may generalize across caregiving configurations even though the specific dyad through which communication operates protectively may differ. This implies that the quality of remote communication, assessed in terms of emotional attunement and reliability, may be at least as clinically important as its frequency; brief interventions targeting emotionally responsive digital communication in migrant families therefore represent a feasible component of family-level mental health support.

As in the preceding sections, the resilience evidence reviewed here is not evenly distributed across caregiving configurations: grandparental involvement and coparenting-harmony pathways derive from urban, parent-present samples (Wu et al., 2025; Xie et al., 2026; Zhu et al., 2024; Luo et al., 2020), while the parent–child communication pathway derives specifically from LBC samples (Wang et al., 2019). Direct comparative testing of these pathways across the two family configurations has not, to our knowledge, been conducted.

## 5 Clinical and nursing implications

Several direct implications for psychiatric and nursing practice with Chinese children and families emerge from the evidence synthesized above. Mental health practitioners should adopt a multigenerational assessment approach when evaluating children presenting with behavioral or emotional difficulties in Chinese family contexts, and this assessment should explicitly distinguish between grandparent-only custodial arrangements (where grandparental caregiving quality is the primary variable of clinical interest) and triadic parent-grandparent coparenting arrangements (where the coparenting relationship itself becomes an additional, independent target of assessment). Standard clinical protocols focused exclusively on the parent–child dyad may systematically miss the intergenerational coparenting dynamics through which much of the risk and resilience in Chinese families is transmitted. Such an approach would ideally capture bidirectional influences between grandparental and parental caregiving subsystems, recognizing that the coparenting relationship functions as a proximal context within which both sets of caregiving behaviors are embedded. Screening instruments assessing coparenting relationship quality, grandparental involvement, and caregiver mental health should be integrated as standard components of child psychiatric assessment in Chinese clinical settings.

From a nursing perspective, there is compelling evidence for family-centered care models that explicitly address intergenerational coparenting dynamics. Chen J. et al. (2024) and Chen S.-M. et al. (2024) specifically recommended parallel intervention sessions for both parents and grandparents, based on evidence of mutual caregiver influence within the coparenting dyad. This recommendation aligns with the broader principle that interventions in Chinese three-generation families are more effective when engaging all relevant coparenting parties simultaneously rather than focusing on individual caregivers in isolation (Liang and Van Leeuwen, 2025). Pediatric and community nurses are particularly well positioned to deliver brief psychoeducational interventions addressing grandparental caregiving roles, coparenting communication, and conflict resolution, given their access to families during routine well-child visits and community health checks. For grandparent-only custodial families (BLBC), where a present coparenting partner is unavailable, nursing interventions may instead need to focus on strengthening grandparental caregiving competence directly and on facilitating emotionally attuned remote parent–child communication, rather than on dyadic coparenting negotiation.

Evidence-based parenting programs offer adaptable frameworks for structured clinical intervention. The Triple P-Positive Parenting Program has demonstrated efficacy across culturally diverse populations, including Chinese families in Hong Kong and Singapore, with significant effect sizes for child behavioral outcomes (*d* = 0.473) and parenting practices (*d* = 0.578) in a systematic meta-analysis of 116 studies (Sanders et al., 2014). A randomized controlled trial in Vietnam demonstrated that a culturally adapted Triple P plus compassion module significantly improved coparenting conflict, parenting behaviors, and child prosocial behaviors between parents and grandparents, with effects sustained at six-month follow-up (Hoang et al., 2022). Contrary to our original framing, direct evidence for Triple P adaptations in mainland Chinese and Hong Kong populations is in fact available and merits explicit discussion. A randomized controlled trial of Group Triple P with 81 Chinese parents in Shanghai found significant improvements in child adjustment problems, parenting practices, parental adjustment, and parenting self-efficacy at post-assessment, with gains maintained at six-month follow-up (Guo et al., 2016). More directly relevant to the intergenerational focus of this paper, a randomized controlled trial of the Grandparent Triple P (GTP) program with 56 Hong Kong Chinese grandparents found a significant decrease in grandchildren’s behavior problems and a significant increase in grandparent self-efficacy following intervention, along with qualitative reports of reduced grandparent role-related stress (Leung et al., 2014). The evidence indicates that both standard Group Triple P and a grandparent-specific adaptation have already demonstrated efficacy in Chinese samples, including a mainland Chinese sample. At the same time, a review of Triple P implementation in China notes that effects for Grandparent Triple P and discussion-group variants have been more limited than for core Group Triple P, and that the program’s strategies may be insufficiently tailored to address grandparental involvement in childrearing and left-behind children’s needs specifically, suggesting that further cultural and structural adaptation—rather than wholesale importation—remains warranted (Liu and Guo, 2020).

Given the strong influence of filial piety norms and Confucian values on intergenerational family dynamics, clinical interventions must be culturally responsive (Tao et al., 2025). Filial piety obligates adult children to defer to parental and grandparental authority even in adulthood, and this norm can generate ambiguity over who holds final decision-making authority when parents and grandparents disagree on child-rearing practices (Yeh and Bedford, 2003). Empirical work on urban Chinese three-generation families indicates that grandparents’ “authoritative resources”—encompassing economic contributions (e.g., housing, financial support), ethical standing rooted in filial obligation, and psychological influence accumulated through the relationship history with their adult children—shape the distribution of power within the intergenerational coparenting relationship, and that grandparental psychological control is associated with lower coparenting relationship quality, with these dynamics differing depending on whether the involved grandparent is maternal or paternal (Chen and Du, 2023). Role ambiguity and authority conflicts between parents and grandparents are embedded within broader cultural scripts that assign high legitimacy to grandparental authority, consistent with the “power and authority” dimension identified as a recurring source of intergenerational role conflict in the scoping review by Bai et al. (2023), and with ethnographic work describing the negotiated, non-institutionalized nature of power-sharing between young mothers and grandparents in urban Chinese childrearing coalitions (Chen, 2016). Mental health and nursing professionals should avoid pathologizing culturally normative grandparenting arrangements and instead work collaboratively with families to negotiate adaptive coparenting roles that honor cultural values while minimizing conflict and child-related harm (Rong and Yuan, 2025; Yuan et al., 2025).

In sum, the clinical and nursing recommendations in this section draw on evidence spanning both custodial (grandparent-only) and triadic (parent-present) caregiving configurations; where the underlying evidence base is configuration-specific, we have flagged this explicitly above to guide practitioners in selecting the intervention approach most appropriate to a given family’s caregiving structure.

## 6 Limitations and future directions

The present synthesis draws predominantly on cross-sectional observational studies, which limits causal inference. Longitudinal designs and randomized trials of intergenerational coparenting interventions tailored to Chinese cultural contexts are needed to establish directionality and to evaluate the efficacy of the recommended clinical approaches. Much of the current evidence derives from either rural LBC samples or urban three-generation households, with limited attention to the heterogeneity of Chinese family configurations across regional, socioeconomic, and migratory contexts. As highlighted throughout this paper, the LBC-focused evidence (Sections 2 and 4’s parent-child communication pathway) and the intergenerational coparenting evidence (Sections 3 and 4’s grandparental involvement and coparenting-harmony pathways) derive from largely non-overlapping samples and have rarely been directly compared within a single study. We also emphasize that this evidentiary separation should not be mistaken for a claim that parental involvement is absent in LBC families; as noted in Section 3, migrant parents frequently remain involved from a distance, and at least one study has modeled this remote involvement jointly with grandparental involvement (Chen J. et al., 2024; Chen S.-M. et al., 2024). Future studies should more systematically incorporate measures of remote/digital parental involvement alongside grandparental involvement and coparenting relationship quality, rather than treating LBC families as caregiving contexts in which parents are simply “absent.” Consequently, it remains unclear whether the risk and resilience mechanisms identified in urban, parent-present, three-generation households generalize to left-behind children—particularly to those whose both parents have migrated and who lack a present coparenting partner altogether—or whether distinct mechanisms operate in each configuration. Future research employing harmonized measures across both rural LBC and urban three-generation household samples, ideally within the same study, would help clarify the extent to which grandparenting quality, coparenting relationship quality, and family resilience operate as configuration-general versus configuration-specific mechanisms. Future research should examine how different coparenting profiles moderate the trajectory of child mental health difficulties over developmental time and should include clinical samples to facilitate direct translation to psychiatric and nursing practice.

## Data Availability

The original contributions presented in the study are included in the article/supplementary material, further inquiries can be directed to the corresponding author.

## References

[ref1] Akezhuoli H. Lu J. Zhao G. Xu J. Wang M. Wang F. . (2022). Mother’s and father’s migrating in China: differing relations to mental health and risk behaviors among left-behind children. Front. Public Health 10:894741. doi: 10.3389/fpubh.2022.894741, 35719620 PMC9203828

[ref2] Bai X. Chen M. He R. Xu T. (2023). Toward an integrative framework of intergenerational coparenting within family systems: a scoping review. J. Fam. Theory Rev. 15, 78–117. doi: 10.1111/jftr.12478

[ref3] Brumariu L. E. (2015). Parent-child attachment and emotion regulation: parent-child attachment and emotion regulation. New Dir. Child Adolesc. Dev. 2015, 31–45. doi: 10.1002/cad.20098 26086126

[ref4] Chen J. (2016). Intimate power: the intergenerational cooperation and conflicts in childrearing among urban families in contemporary China. J. Chin. Sociol. 3:18. doi: 10.1186/s40711-016-0037-y

[ref5] Chen J. Du P. (2023). Grandparents' authoritative resources and intergenerational coparenting relationship in Chinese urban families: differences between maternal and paternal grandparents. Chin. J. Sociol. 43, 54–78.

[ref6] Chen S.-M. Gao L. Lin S.-H. Zhang Y. (2024). Relationship between parental involvement and emotional well-being in Chinese left-behind children: a moderated mediation model. Fam. Relat. 73, 1346–1361. doi: 10.1111/fare.12905, 40046247

[ref7] Chen J. Zhou X. Bai X. (2024). Associations between parents’ and grandparents’ depressive symptoms, intergenerational coparenting relationships, and (grand)parenting behaviors: An actor–partner interdependence mediation model. Fam. Process 63, 1409–1428. doi: 10.1111/famp.12916, 37434285

[ref8] Feinberg M. E. (2003). The internal structure and ecological context of coparenting: a framework for research and intervention. Parenting 3, 95–131. doi: 10.1207/S15327922PAR0302_01, 21980259 PMC3185375

[ref9] Guo M. Morawska A. Sanders M. R. (2016). A randomized controlled trial of group triple P with Chinese parents in mainland China. Behav. Modif. 40, 825–851. doi: 10.1177/0145445516644221, 27091951

[ref10] He T. Wu D. Jiang Y. Tang Y. Zhou Q. Wu Q. . (2024). Coparenting relationship, parenting self-efficacy, and child adjustment in Chinese mother–grandmother coparenting families: grandmother role difference. Fam. Relat. 73, 1086–1106. doi: 10.1111/fare.12928

[ref11] Hoang N.-P. T. Kirby J. N. Haslam D. M. Sanders M. R. (2022). Promoting positive relationship between parents and grandparents: A randomized controlled trial of group triple P plus compassion in Vietnam. Behav. Ther. 53, 1175–1190. doi: 10.1016/j.beth.2022.06.002, 36229115

[ref12] Leung C. Sanders M. Fung B. Kirby J. (2014). The effectiveness of the grandparent Triple P program with Hong Kong Chinese families: a randomized controlled trial. J. Fam. Stud. 20, 104–117. doi: 10.1080/13229400.2014.11082000

[ref13] Li J. Chen Y. Zhao X. Xue E. (2026). Investigating the mental health of left-behind children in China’s rural areas: a systematic review approach. Int. J. Ment. Health Promot. 28, 1–10. doi: 10.32604/ijmhp.2026.073286

[ref14] Li Z. Daud M. N. Abdul Ghani M. S. B. Ashari A. (2026). Intergenerational relational dynamics in co-parenting families: a cross-sectional analysis through a systemic theoretical lens. Front. Psychol. 17:1748675. doi: 10.3389/fpsyg.2026.1748675, 42099716 PMC13143887

[ref15] Li X. Xie J. Song Y. (2016). Grandparents-parents coparenting and its relationship with maternal parenting stress and children’s behavioral problems. Chin. J. Spec. Educ. 4, 71–78.

[ref16] Li Y. Xie J. Song Y. Hu F. Zhang X. (2019). The relationship between parenting styles practiced by grandparents and children's emotional and behavioral problems. J. Child Fam. Stud. 28, 1899–1913. doi: 10.1007/s10826-019-01415-7, 37006465 PMC10065465

[ref17] Liang R. Van Leeuwen K. (2025). Distant mothering, grandparenting, intergenerational coparenting relationship, and child adjustment: Evidence from Chinese families with young left-behind children. Front. Psychol. 16:1660162. doi: 10.3389/fpsyg.2025.1660162, 41479966 PMC12753985

[ref18] Liu Y. Guo M. (2020). Triple P—positive parenting program: a review and implications for its social and cultural acceptability in China. J. Psychol. Sci. 43, 1376–1383.

[ref19] Luo Y. LaMonica S. Xu S. Cui M. (2020). Grandparent involvement and preschoolers' social adjustment in Chinese three-generation families: examining moderating and mediating effects. Child Youth Serv. Rev. 118:105457. doi: 10.1016/j.childyouth.2020.105457

[ref20] Minuchin P. (1988). “Relationships within the family: a systems perspective on development,” in Relationships within Families: Mutual Influences, eds. R. A. Hinde and J. Stevenson-Hinde. (Oxford: Clarendon Press), 7–26.

[ref21] Ran H. Chang W. Xu C. Che Y. Fang D. Chen L. . (2024). Depressive disorders in Chinese left-behind children and adolescents from Yunnan province: prevalence and association with self-harm behaviors. Arch. Public Health 82:161. doi: 10.1186/s13690-024-01393-3, 39294695 PMC11409802

[ref22] Rong Y. Yuan G. F. (2025). Family-centered mental health care for left-behind children in Chinese ethnic minority concentrated areas: a psychiatric nursing framework for rural education settings. Front. Public Health 13:1709606. doi: 10.3389/fpubh.2025.1709606, 41458406 PMC12738909

[ref23] Sanders M. R. Kirby J. N. Tellegen C. L. Day J. J. (2014). The triple P-positive parenting program: a systematic review and meta-analysis of a multi-level system of parenting support. Clin. Psychol. Rev. 34, 337–357. doi: 10.1016/j.cpr.2014.04.003, 24842549

[ref24] Tao Y. Lu Y. Shi W. Yuan G. F. (2025). Multidimensional diagnostic features and intervention pathways for youth mental disorders in China’s involution society. Asian J. Psychiatr. 108:104531. doi: 10.1016/j.ajp.2025.104531, 40382979

[ref25] Tong L. Yan Q. Kawachi I. (2019). The factors associated with being left-behind children in China: multilevel analysis with nationally representative data. PLoS One 14:e0224205. doi: 10.1371/journal.pone.0224205, 31710607 PMC6844451

[ref26] Wang D. Chen H. Chen J. Ye H. Chen X. Ye L. . (2023). A comparative study of mental health status among left-behind, migrant, and local adolescents in China. J. Affect. Disord. 324, 521–528. doi: 10.1016/j.jad.2022.12.134, 36586615

[ref27] Wang F. Lin L. Xu M. Li L. Lu J. Zhou X. (2019). Mental health among left-behind children in rural China in relation to parent-child communication. Int. J. Environ. Res. Public Health 16:1855. doi: 10.3390/ijerph16101855, 31130670 PMC6572381

[ref28] Wang Y. Lu S. Fu L. Du N. Xu D. (2024a). Voices behind images: exploring left-behind children's lives with grandparents through Photovoice. Br. J. Soc. Work. 54, 1867–1888. doi: 10.1093/bjsw/bcad264

[ref29] Wang Y. Wu Q. Wang C. Guan Y. (2024b). Research review: Grandparental care and child mental health—a systematic review and meta-analysis. J. Child Psychol. Psychiatry 65, 568–586. doi: 10.1111/jcpp.13943, 38171720

[ref30] Wu J. Zhu J. Lei Y. Shi L. (2025). Effects of grandparents’ involvement on young children’s resilience: mother’s parenting stress and family strength as mediators. Psicol. Reflex. Crit. 38:33. doi: 10.1186/s41155-025-00370-1, 41258607 PMC12630510

[ref31] Xie Z. Zhang X. Zhang Y. Zhang Y. Luo J. Xiao X. (2026). Patterns of Intergenerational co-parenting relationships in Chinese families with infants and toddlers: a latent profile analysis. J. Adv. Nurs. 82, 7337–7351. doi: 10.1111/jan.70379, 41249889

[ref32] Yeh K. Bedford O. (2003). A test of the dual filial piety model. Asian J. Soc. Psychol. 6, 215–228. doi: 10.1046/j.1467-839X.2003.00122.x

[ref33] Yu Y. Li S. Chen Y. (2025). Measuring child poverty in rural China: evidence from households with left-behind and non-left-behind children. China Econ. Rev. 90:102354. doi: 10.1016/j.chieco.2025.102354

[ref34] Yuan G. F. Rong Y. Shi W. (2025). Parental mental health support for families of children with psychiatric disorders: a framework for service system development in China. Asian J. Psychiatr. 116:104809. doi: 10.1016/j.ajp.2025.104809, 41485446

[ref35] Zhao F. Wu H. Li Y. Zhang H. Hou J. (2022). The association between coparenting behavior and internalizing/externalizing problems of children and adolescents: a meta-analysis. Int. J. Environ. Res. Public Health 19:10346. doi: 10.3390/ijerph191610346, 36011980 PMC9407961

[ref36] Zhu J. Xiang S. Li Y. (2024). Mothers’ perceived co-parenting and preschooler’s problem behaviors: the mediating role of maternal parenting stress and the moderating role of family resilience. Psychol. Res. Behav. Manag. 17, 891–904. doi: 10.2147/PRBM.S451870, 38476351 PMC10929244

